# P-43. A Single Composite Peptide Vaccine Comprising Conserved Influenza Epitopes Formulated with ALFQ Adjuvant Induced Broadly Reactive Antibodies to Human, Avian, and Swine Influenza Viruses

**DOI:** 10.1093/ofid/ofae631.250

**Published:** 2025-01-29

**Authors:** Nimisha Rikhi, Clara J Sei, Mangala Rao, Kellie Kroscher, Gary Matyas, Richard F Schuman, Kevin Muema, Camille Lange, Aba Assiaw-Dufu, Carl R Alving, Gerald W Fischer

**Affiliations:** Longhorn Vaccines & Diagnostics, LLC, Gaithersburg, Maryland; Longhorn Vaccines & Diagnostics, LLC, Gaithersburg, Maryland; Military HIV Research Program, Silver Spring, Maryland; Longhorn Vaccines & Diagnostics, LLC, Gaithersburg, Maryland; Military HIV Research Program, Silver Spring, Maryland; Antibody and Immunoassay Consultants, Rockville, Maryland; Longhorn Vaccines & Diagnostics, LLC, Gaithersburg, Maryland; Henry M Jackson Foundation for the Advancement of Military Medicine, Bethesda, Maryland; Longhorn Vaccines & Diagnostics, LLC, Gaithersburg, Maryland; Walter Reed Army Institute of Research, Silver Spring, Maryland; Longhorn Vaccines & Diagnostics, LLC, Gaithersburg, Maryland

## Abstract

**Background:**

Reassortant influenza strains containing genes from human, avian, and/or swine origin, have led to major outbreaks, such as the 2009 swine flu pandemic. The ongoing global spread of highly pathogenic avian influenza (HPAI) A/H5N1 virus among poultry highlights the risk of zoonotic transmission leading to pandemics. A universal influenza vaccine that generates broadly neutralizing antibodies against influenza viruses is crucial for preventing future pandemics. Previous studies with our unconjugated composite peptide vaccine (two-peptide combination), which targets multiple highly conserved epitopes on influenza virus, generated broadly reactive antibodies to human and avian strains, including HPAI A/H5N1 viruses. A single peptide vaccine approach offers formulation and scale-up advantages. Here, we demonstrate that a novel, unconjugated, single composite peptide vaccine comprising highly conserved epitopes of hemagglutinin (HA), neuraminidase (NA), and matrix (M1/M2/M2e), and a universal T cell epitope, formulated with the highly potent adjuvant Army Liposomal Formulation with QS21 (ALFQ), induced broadly reactive antibodies to human, avian, and swine influenza strains.

**Methods:**

ICR mice were immunized intramuscularly on days 0, 21, and 35 with 2μg of composite influenza peptide vaccine LHNVD-110, formulated with ALFQ adjuvant. Isotype-specific IgG titers to peptides and influenza A (H1N1pdm09, H3N2, HPAI H5N1) and B (Victoria, Yamagata) viruses were analyzed by ELISA. Neutralizing titers were determined using the microneutralization assay and inhibition of HA using the HAI assay.

**Results:**

Robust serum IgG1 antibodies to human, avian, and swine influenza A and B viruses were induced. Post-immunization serum demonstrated neutralization and HA inhibition of Group 1 and 2 influenza viruses.

**Conclusion:**

An unconjugated single composite peptide vaccine comprising highly conserved HA, NA, and matrix epitopes, formulated with ALFQ adjuvant, generated broadly reactive antibodies to human, avian, and swine influenza strains and neutralized seasonal and pandemic strains. This single composite peptide vaccine provides a cost-effective, and easily scalable strategy towards a universal influenza vaccine to combat future pandemics.

**Disclosures:**

**All Authors**: No reported disclosures

